# High efficiency and scalable fabrication of fresnel zone plates using holographic femtosecond pulses

**DOI:** 10.1515/nanoph-2022-0112

**Published:** 2022-05-24

**Authors:** Zhipeng Wang, Lan Jiang, Xiaowei Li, Shuai Gao, Shipeng Zhou, Yang Liu, Lingling Huang, Jiangang Lu, Jiangang Yin

**Affiliations:** Laser Micro/Nano Fabrication Laboratory, School of Mechanical Engineering, Beijing Institute of Technology, Beijing 100081, China; Beijing Institute of Technology Chongqing Innovation Center, Chongqing, 401120, China; School of Optics and Photonics, Beijing Institute of Technology, Beijing 100081, China; Han’s Laser Technology Centre, Shennan Ave No. 9988, Nanshan District, Shenzhen, Guangdong, 518057, China

**Keywords:** fresnel zone plate, fresnel zone plate array, holographic femtosecond processing, petal-like zone plate, ultrathin binary optics

## Abstract

To meet the growing demand for photonic integration and device miniaturization, planar diffractive Fresnel zone plates (FZPs) are widely applied in integrated optical systems. However, challenges remain in fabricating FZPs with high efficiency and satisfying the requirement for cross-scale fabrication. This paper details a high efficiency method for fabricating ultrathin FZPs of different scales on metal films by using holographic femtosecond lasers. The FZPs are split into a series of element patterns that are printed in order by using corresponding modulated femtosecond pulses. The fabricated FZPs are spliced by the printed element structures with no FZP size limitation in theory. FZPs with an area varying across three orders of magnitude are presented to demonstrate the capability of cross-scale fabrication. The fabricated FZPs possess an excellent broadband focusing and imaging ability in the visible spectrum. Furthermore, the fabrication of other functional ultrathin lenses, such as axial multifocal zone plates, petal-like zone plates, and FZP arrays, is described, revealing the wide potential for the flexible and scalable fabrication method in on-chip integrated optical systems.

## 1 Introduction

Refractive optics such as lens, grating, axicon, and microlens arrays are widely used in optical systems and daily life, but their bulky size and poor installation flexibility render them incapable of meeting the growing demand for miniaturization in integrated optical systems. Planar diffractive optical devices, however, have attracted broad attention because they are ultrathin, light weight, and easy to integrate [1–5]. The Fresnel zone plate (FZP) is a typical diffractive optical device that consists of alternate concentric rings of various thicknesses (phase type) [6, 7] or optical transmittances (amplitude type) [8, 9]. The light beams transmitted or reflected by FZPs interfere constructively at the designed points, and hence, they are generally used in optical systems as the refractive lens for focusing and have been extensively applied in hybrid optics, microsensors, optical tweezers, and interconnections [10–13]. Similar to other diffractive optical devices, FZPs are commonly fabricated using high-accuracy processing methods, such as photolithography [14], electron-beam lithography [15], or focused ion beam etching [16]. However, these methods require a multistep manufacturing process, the processing of masks, or rigorous environmental conditions, which makes processing time-consuming and costly, thus limiting the practical application of FZPs and their integration with other optical devices.

Femtosecond lasers have attracted broad attention in micro–nano fabrication because of their unique ultrashort pulse duration, extremely high peak intensity, and material universality [17–21] as well as their inherently flexible and maskless laser processing. Because of the ultrashort pulse duration, the laser pulse ends before the energy is transferred from the excited electrons to the lattice, which minimizes the thermal effect. The small heat-affected zone increases the processing accuracy of femtosecond lasers compared with that of other laser beams, which makes femtosecond laser processing a flexible and high-precision tool for fabricating micro–nano structures [19, 22]. Various FZPs based on different material systems have been fabricated through femtosecond laser direct writing. These include surface FZPs fabricated on polymethyl methacrylate, polycarbonate, fused silica, and the end facet of multimode fibers by using laser ablation [23–26]; volumetric FZPs fabricated in borosilicate glass, fused silica, and sapphire by using laser-induced localized refractive index change or void generation [27–30]; and nonlinear FZPs fabricated in lithium niobate crystal by using laser-induced quadratic susceptibility erasion [31]. Furthermore, ultrathin FZPs fabricated on film materials such as graphene oxide [32] or metal [33] with a nanometric thickness are gaining attention because of their flexibility and compact size and their ability to be transferred to other substrates or the surface of optical elements without functional deterioration, which is beneficial for optical microsystem integration.

Although femtosecond laser direct writing has simplified the FZP fabrication process, single-focus processing makes it still take a long time to fabricate FZPs with large scale or FZP arrays. Employing galvanometric scanning systems to increase the scanning speed of the laser focus has proved to be an effective method of improving fabrication efficiency [24, 32], but the low numerical aperture (NA) of the f-theta lens in the scanning system limits processing accuracy. Holographic femtosecond laser processing based on spatial light modulators (SLMs) is another way to improve fabrication efficiency [34]. By loading corresponding computer-generated holograms (CGHs) into SLMs, a single laser focus can be modulated to predesign intensity distributions, such as multifocal arrays or patterned optical fields, with high flexibility and controllability. Diverse functional microdevices, including microfluidic filters [35], biconical microtubes [36], waveguides [37], microlens arrays [38], and holograms [39], have been fabricated using holographic femtosecond lasers. There is also report about rapidly fabricating FZPs using a patterned laser field consisting of concentric circles [40]. However, because of field-of-view limitations in objective lenses and the spatial dispersion of peripheral focal spots, the overall size of microdevices printed using modulated optical fields is generally restricted to between tens of microns and hundreds of microns. Thus, developing an effective method for the scalable fabrication of microdevices of different sizes is desirable.

In this study, a high-efficiency method for fabricating binary amplitude FZPs of different scales on gold film using holographic femtosecond laser pulses is proposed. The FZP patterns are split into a series of elements according to their size. The elements are then printed on Au film by dynamically modulating the femtosecond pulses and, finally, stitching them together to form complete FZPs. Because of the splitting and stitching process, this method, theoretically, has no limitations in terms of FZP size. FZPs with an area varying across three orders of magnitude were fabricated using the same processing system. The fabricated FZPs exhibited a broad working band in the visible spectrum, which is highly desirable in multiwavelength optical imaging and communications. Apart from FZPs, axial multifocal zone plates, various petal-like zone plates (PZPs), and FZP arrays were fabricated to demonstrate the high flexibility and application potential of this proposed method for fabricating functional ultrathin lenses.

## 2 Materials and methods

### 2.1 Experimental setup and materials

**Figure 1:**
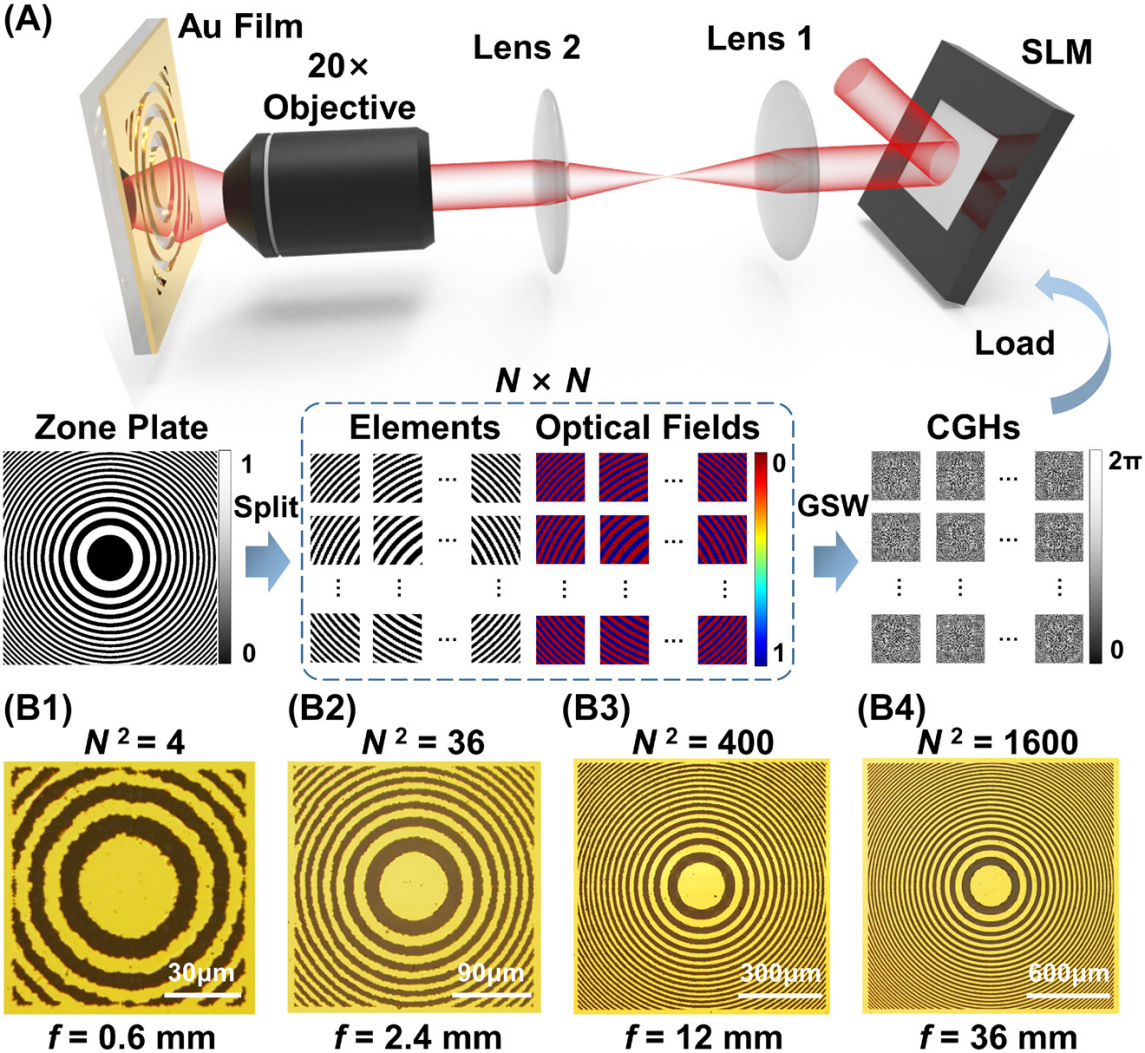
Scheme and experimental results of the FZP fabrication process using holographic femtosecond laser pulses. (A) Scheme of the splitting–stitching method for fabricating FZPs. The calculated FZP pattern is split into *N* ^2^ elements, which are regarded as target modulation optical fields. By dynamically loading the calculated CGHs onto the SLM and moving the Au film synchronously, the element structures printed using modulated pulses are stitched together, constituting complete FZPs. Microscopy images of the fabricated FZPs with areas of (B1) 8.1 × 10^3^, (B2) 7.29 × 10^4^, (B3) 8.1 × 10^5^, and (B4) 3.24 × 10^6^ μm^2^, demonstrating the cross-scale fabrication capability of the proposed method.

The holographic femtosecond laser processing setup is presented in Figure 1A. The femtosecond laser pulses (35 fs pulse duration, 1 kHz repetition rate, 800 nm central wavelength) from a commercial Ti:sapphire laser regenerative amplifier system (Spitfire Ace-35 F, Spectra Physics, USA) with a diameter of 8 mm were modulated using a reflective liquid-crystal-on-silicon SLM (8 μm pixel pitch, 1920 × 1080 px resolution, Pluto-NIR, Holoeye, Germany). After being delivered through a 4 *f* (*f* = 600 mm) optical system, the modulated pulses were focused using an objective lens (20 × /NA = 0.45), forming the patterned optical fields at the focal plane. The pulse energy and number were adjusted by using a neutral density attenuator and mechanical shutter, respectively. The 30 nm-thick Au film was deposited on the SiO_2_ substrate with thickness of 500 μm, which was mounted on a six-axis moving stage (M-840.5DG, PI, USA). The sample stage, shutter, and SLM were synchronously controlled using a home-built software.

### 2.2 Sample characterization

The morphology of the fabricated FZPs was characterized using an industrial microscope (BX53, Olympus, Japan), field-emission scanning electron microscope (SU8200, Hitachi, Japan), and atomic force microscope (Dimension Edge PSS, Bruker, Germany). For the optical property characterization, a supercontinuum laser source (SuperK EXTREME, NKT Photonics, Denmark) was employed to provide laser beams with wavelengths of 470, 532, and 633 nm. The cross-sectional intensity distributions of the diffracted optical fields of the zone plates were magnified through an objective (100 × /NA = 0.9 or 50 × /NA = 0.6) and lens (*f* = 100 mm), which were captured using a charge-coupled device (Lm11059C, Lumenera, Canada). By adjusting the position of zone plates along the optical axis, intensity distributions at different axial positions were obtained.

## 3 Results and discussion

### 3.1 Illustration of the splitting–stitching method for fabricating FZPs

The scheme of the proposed method is presented in Figure 1A. The *M* × *M* px FZP pattern consists of alternate concentric rings of white or black pixels. The rings with white pixels correspond to the transparent zones with a transmittance of 100% in the FZPs, and the materials in these zones must be removed through laser ablation during the fabrication process. The rings containing black pixels are opaque zones with zero transmittance, corresponding to the remaining metal zones in the fabricated FZPs. The FZP pattern is then split into *N* ^2^ elements, containing *M*/*N* × *M*/*N* px. Each element is regarded as a target optical field, with the white pixels corresponding to focal points and the black pixels corresponding to the zero-intensity background. The CGHs of every target optical field are calculated using the weighted Gerchberg–Saxton (GSW) algorithm. During the fabrication of the FZPs, pattern structures corresponding to the FZP elements are printed individually by dynamically modulating the femtosecond pulses through loading different CGHs onto the SLM. The Au film is ablated completely under the irradiation of patterned optical fields, exposing the transparent SiO_2_ substrate. By programmatically moving the sample stage, the printed element structures are stitched together, constructing the complete FZP.

The radius of the concentric rings in a FZP is defined as

(1) $$R_{\text{m}}=\sqrt{mf\lambda }$$

where *m* is the number of the *m*th zone, and *f* and *λ* are the focal length and working wavelength of the FZP, respectively [29]. Figure 1B1–4 presents the microscopy images of the fabricated FZPs designed at *λ* = 633 nm, with the focal lengths of 0.6, 2.4, 12, and 36 mm and pixel numbers of 60 × 60, 180 × 180, 600 × 600, and 1200 × 1200 px, respectively. During the splitting process, each element split from the FZP patterns contains 30 × 30 px, corresponding to a 45 × 45 μm^2^ printed structure with a pixel size of 1.5 μm. Therefore, the element numbers of the four FZPs are 4, 36, 400, and 1600, respectively. Because the thin gold film is sensitive to the thermal effect, single laser pulse was used to process every element of the FZPs to alleviate the extra thermal effect and overexposure. In addition, in order to completely remove the Au film in the ablation zone, the elements were printed by using holographic pulses with energy of approximately 33 μJ. Under such pulse energy, the size of processed holes on the Au film by the optical spots of modulated optical fields was slightly larger than the pixel size of zone plates (1.5 μm), which ensured that there was no residual material between the processed holes and made the ablated area continuous. The areas of the presented FZPs are 8.1 × 10^3^, 7.29 × 10^4^, 8.1 × 10^5^, and 3.24 × 10^6^ μm^2^, respectively, demonstrating a cross-scale of three orders of magnitude.

**Figure 2:**
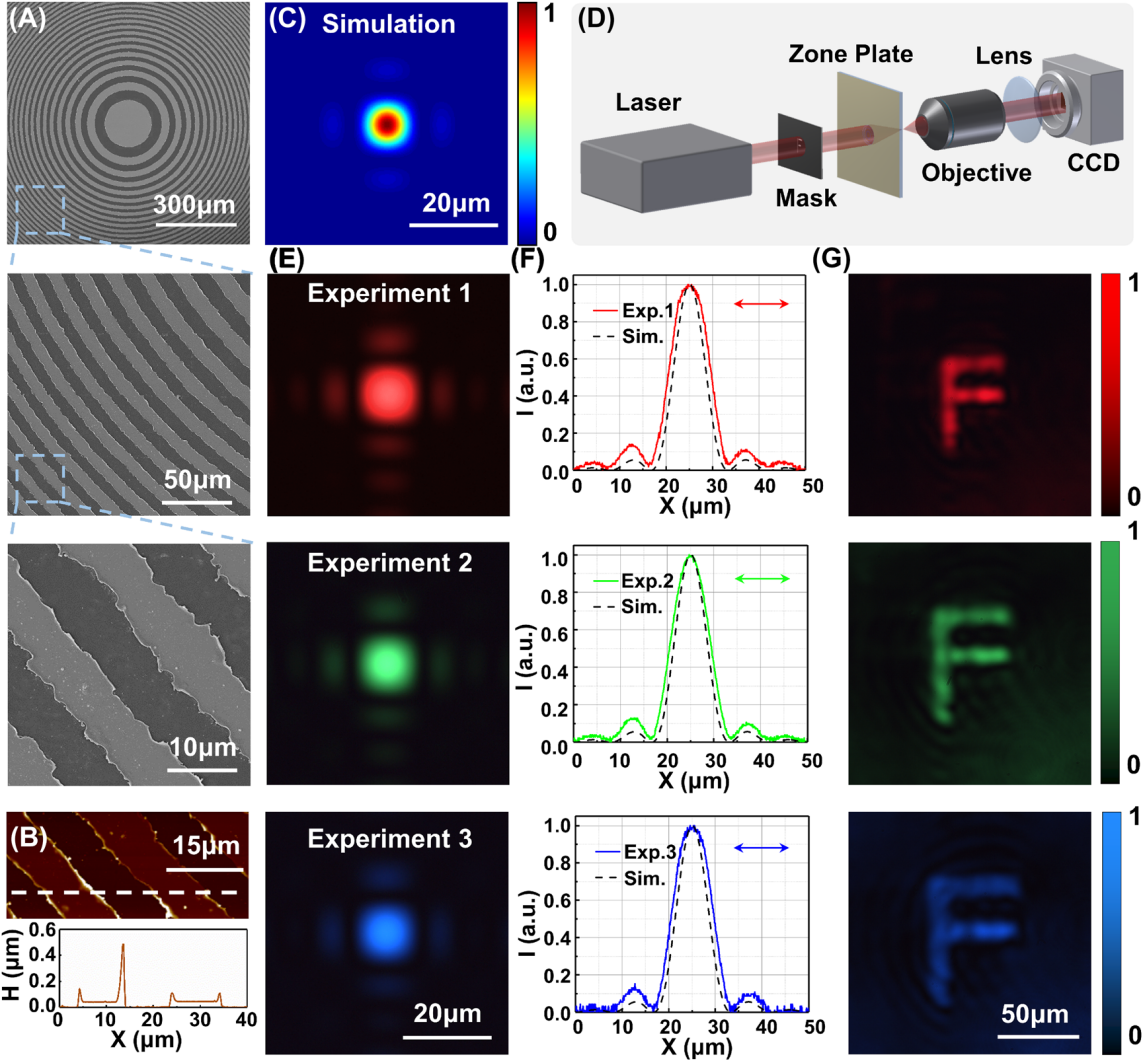
Morphology and optical property characterization of the fabricated FZP. (A) SEM images of the fabricated FZP with *f* = 12 mm. (B) Atomic force microscopy image of the metal rings. The bottom graph depicts the height profile along the white dashed line. (C) Simulated intensity distribution at the focal plane of the FZP. (D) scheme of the optical system for the optical property characterization. (E) measured intensity distributions at focal planes under the irradiation of laser beams at 470, 532, and 633 nm. (F) Corresponding normalized intensity profiles plotted along the central cross section of the focal spots. (G) mask containing a hollow pattern forming the microletter “F” imaged using the FZP at different laser wavelengths.

Figure 2A Displays the scanning electron microscopy (SEM) images of the fabricated FZP with *f* = 12 mm. As illustrated in the microscopy and SEM images in Figures 1B and 2A, the metal film of the ablation zone was completely removed, and the border of the metal rings were serrated because of the pixelation of the FZP pattern and interference between adjacent spots in the modulated optical fields. The outermost zone width (*d*_r_) is a crucial parameter of the FZPs. Under the focusing condition of the objective lens (20×, NA = 0.45) used in the holographic femtosecond laser processing setup, the smallest pixel size of the printed structure that can be achieved by the fabrication method depends on the smallest separation between adjacent optical spots in the modulated optical fields. The smallest separation can be calculated as *d*_separation_ = *λf*_OL_/*W*_pupil_, where *λ* is the wavelength of femtosecond laser, *f*_OL_ is the focal length of the objective lens, and *W*_pupil_ is the side length of the CGH [41]. According to the parameters of the processing setup (*λ* = 800 nm, *f*_OL_ = 9 mm, W_pupil_ = 8 mm), the smallest separation between adjacent optical spots is *d*_separation_ = 900 nm. But due to the phase mismatches of the modulated optical fields in the holographic spatial shaping, random interference will occur between the adjacent optical spots, which make the intensity distribution of modulated optical fields become inhomogeneous and reduces the quality of the printed structures. In order to weaken the random interference between the adjacent optical spots, the interval between the adjacent optical spots can be set to be larger than the smallest separation *d*_separation_ [41]. In our work, the interval is set as 1.5 μm, so the pixel size of the fabricated FZPs is 1.5 μm. In the extreme case, the width of outermost zone just contains only one pixel. Therefore, the resolution limit of *d*_r_ is about 1.5 μm. Furthermore, the edges of some of the metal rings curled because of the thermal stress formed during the laser ablation, separating the Au film around the ablation zone from the SiO_2_ substrate. The curling of the edges was also verified through atom force microscopy and the cross-section profile in Figure 2B. The edges of the metal rings were higher than the center of the rings. Apart from the recast layer, this height difference is mainly attributable to the separation of the metal film from the substrate.

Figure 2C Depicts the simulated intensity distribution at the focal plane of the FZP under the irradiation of a 633 nm laser beam. To characterize the optical properties of the fabricated FZP, an imaging system with tunable laser wavelength in the visible spectrum was built to measure the focusing and imaging capability (Figure 2D). Because the wavelengths of 470, 532, and 633 nm are corresponding to the blue, green, and red lights in the visible spectrum, respectively, laser beams of these wavelengths were selected as representative laser sources. The measured optical intensity distributions at the focal planes are shown in Figure 2E. Because of the variation of working wavelength, the focal length of the FZP was changed to 14.28 mm for 532 nm laser beam and 16.16 mm for 470 nm laser beam. Bright focal spots were observed in the captured images of the three wavelengths, demonstrating the broadband focusing ability of the fabricated FZP. In addition, the intensity distributions around the foci were consistent with the simulation results depicted in Figure 2C. This consistency was also indicated by the normalized intensity profile in Figure 2F plotted along the cross section of the focal spots, from which it can be found that the full-width at half-maximum (FWHM) of the focal spot is about 10 μm. The *d*_r_ of the fabricated FZP with *f* = 12 mm shown in Figure 2A is about 8.5 μm. Thus, the FWHM of the focal spot can be calculated as *d*_FHWN_ = 1.22*d*_r_ ≈ 10.4 μm [42], which is consistent with the values measured directly from the normalized intensity profiles of focal spots.

The focusing efficiencies of the fabricated FZP were also measured, where the efficiency was defined as the ratio of power measured at the focal spot to the total incident laser power. The measured focusing efficiencies were 3.61%, 3.02%, and 4.17% under the wavelengths of 470, 532, and 633 nm, respectively. For the calculated FZP pattern whose normalized transmittance difference between the transparent zones and the opaque zones was 1, the theoretical efficiency was about 7.84%, which was calculated as the ratio of the power integration of the focal spot to the incident power. In fact, the transmittance of SiO_2_ substrate in the visible spectrum (400–700 nm) is about 0.86 and remains approximately constant with the variation of wavelength; while the transmittance of Au film is in the range of 0.1–0.3 and shows a certain degree of wavelength dependence (Figure S1, Supplementary Material). The absorption of incident light by the SiO_2_ substrate and the transmitting of residual light through Au film will reduce the focusing efficiency of the fabricated FZP. Therefore, the laser wavelength will affect the focusing efficiency of the FZP, as well as the focal length. Considering the optical transmittances of SiO_2_ substrate and Au film, the calculated efficiencies were 4.08%, 3.38%, and 4.83% for the wavelengths of 470, 532, and 633 nm, respectively (Figure S1, Supplementary Material). The deviation between the calculated results and the measured results was reduced when the transmittances of materials were taken into account, and they were both in inverse proportion to the transmittance of Au film at the corresponding wavelength. Apart from the optical transmittances of materials, the processing errors, such as the dislocation of structure elements during stitching, the serrated border of the metal rings and the separation of the metal rings’ edges from the substrate, will cause the loss of focusing efficiency. Besides the main focus at the focal plane, there are subsidiary foci in the optical axis of the FZP, which were also detected by translating the FZP along the optical axis (Figure S2, Supplementary Material). Furthermore, the imaging properties were verified by inserting an aluminum foil mask in front of the FZP. The mask contained a hollow pattern forming the microletter “F” created through laser direct writing. The imaging results under different laser wavelengths are presented in Figure 2G, and the clear images of “F” indicate the high-quality imaging capability of the FZP. Because the residual light transmitting through Au film will form the background noise, the clarity of imaging results of FZP can be related to the wavelength. However, due to the transmittance difference in the visible spectrum is not large; the clarity of imaging results under different laser wavelengths shown in Figure 2F has no significant difference. The magnification of FZPs depends on the ratio of the focal length to the object distance, and the focal length is inversely related to the laser wavelength. Therefore, if the distance between the mask and FZP remains unchanged, the image size of the “F” increases with a decreasing laser wavelength.

### 3.2 Fabrication of axial multifocal zone plates

**Figure 3:**
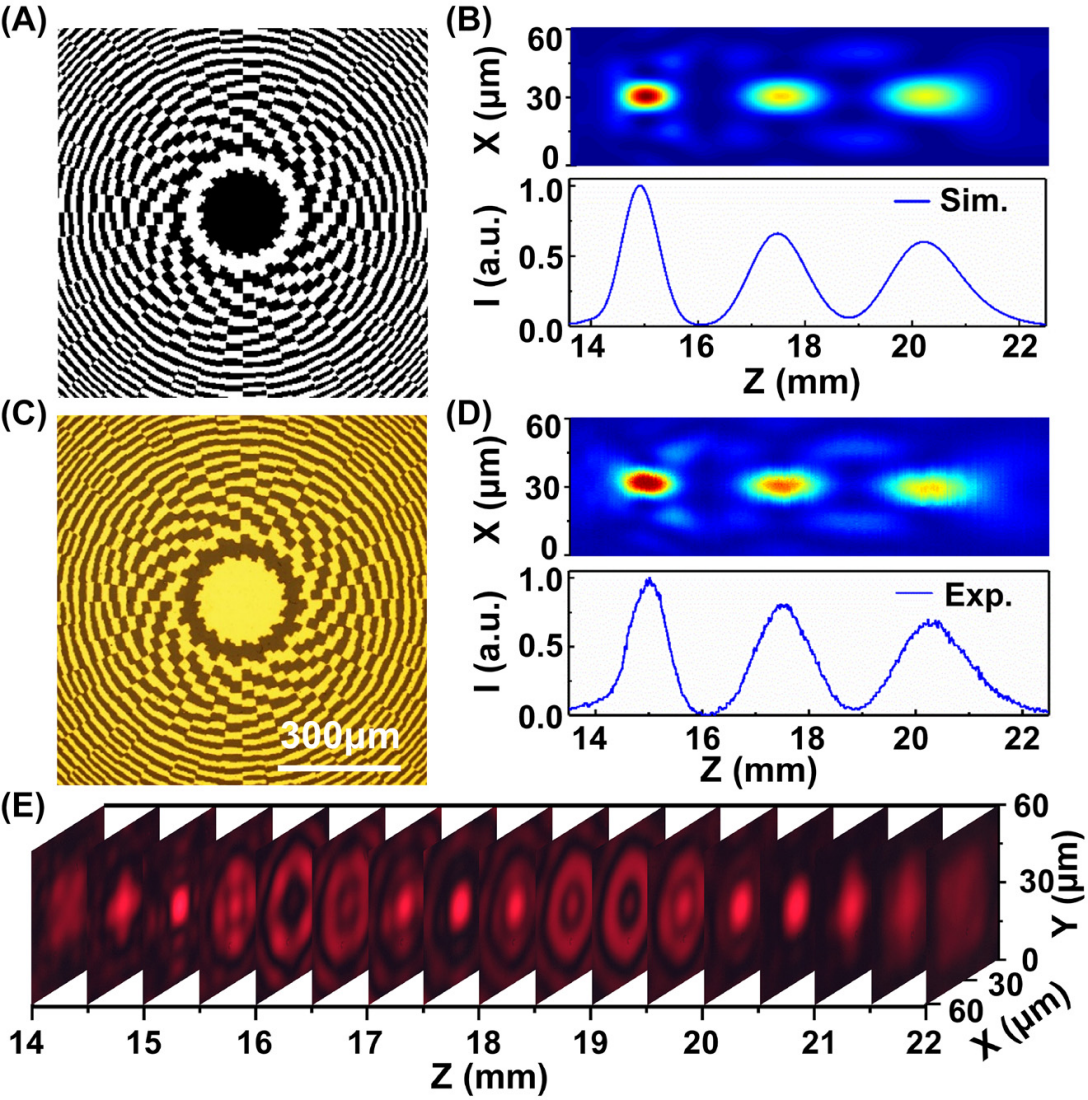
Demonstration of the axial multifocal zone plate. (A) Axial multifocal zone plate (600 × 600 px) developed from three FZPs with focal lengths of 15, 17.5, and 20 mm at a laser wavelength of 633 nm. (B) Simulated longitudinal intensity distribution in the *XZ* plane and the normalized intensity profile along the *Z*-axis of the diffracted optical fields through the multifocal zone plate. (C) Microscopy image of the fabricated multifocal zone plate. (D) measured longitudinal intensity distribution and the corresponding normalized intensity profile. (E) measured intensity distributions in different *XY* planes along the optical axis.

Lenses with designable axial multifoci are highly desirable in three-dimensional imaging, laser fabrication, and optical tweezer technology [43–45]. A zone plate with axial multifoci was designed [46] and fabricated using the splitting–stitching method. The 600 × 600 px axial multifocal zone plate is presented in Figure 3A and consists of 48 sectors with the same center angle, which are periodically filled with the corresponding parts of three different FZPs with focal lengths of 15, 17.5, and 20 mm at a laser wavelength of 633 nm. The specific design process is described in the Supplementary Material. Figure 3B depicts the calculated intensity distribution in the *XZ* plane and the normalized intensity profile along the *Z*-axis. Three foci are distributed along the optical axis, and the volume of the focus spots increases and the peak intensity decreases as the diffracted optical field propagates. Moreover, the positions of the peak intensity of the three foci deviate a little from the designed focal lengths, which can be attributed to the interaction between the different FZPs in the diffracted light.

The fabricated multifocal zone plate on the Au film was composed of 400 element structures (Figure 3C). To measure the intensity distributions of the optical field in the *XZ* plane diffracted by the multifocal zone plate, a series of intensity distributions in the *XY* plane were captured by translating the zone plate along the optical axis with a step of 0.02 mm. The intensity distribution in the *XZ* plane and the corresponding normalized intensity profile were reconstructed and plotted by extracting and sequentially assembling the intensity profiles along the *X*-axis of the focal spots in different *XY* planes (Figure 3D). The intensity distributions in the different *XY* planes along the optical axis are depicted in Figure 3E. The intensity distribution and normalized intensity profile reveal that the intensity distributions and positions of the three foci are in good agreement with the simulation results. The distortion of the foci in the reconstructed result was caused by an alignment error in the optical elements and vibration of the laser beam. Furthermore, the imaging capability of the multifocal zone plate was also detected (Figure S4, Supplementary Material). The images captured at the three focal planes are not as clear as the results from using a single FZP (Figure 2G), and the imaging background contains some noise. The reduction in imaging clarity was mainly caused by the incompleteness of the FZP and the interaction between the foci.

### 3.3 Fabrication of petal-like zone plates

In addition to converging the light beams into a single point at the focal plane, the focused intensity distributions can be modulated into patterns by modifying the zone geometry of the FZP for extensive practical applications. PZPs are a typical deformation of FZPs, consisting of spiral or annular petal-like zones with corresponding focused intensity distributions that form polygon-like or star-like patterns, respectively [47–49]. Furthermore, spiral PZPs can be employed to generate optical vortices [50].

The transmittance function of PZPs in the polar coordinate [47] is calculated as follows:

(2) $$T\left(r,\theta \right)=\text{exp}\left\{-,i,\pi ,\frac{{\left[r,-,r_{0},+,\alpha ,R,{\left(\text{cos},\left(K,\theta \right)\right)}^{m}\right]}^{2}}{\lambda f},+,i,l,\theta \right\}$$

where *r*_0_, *λ*, *f*, and *R* are the original side length, working wavelength, focal length, and side length of the PZP, respectively, and *α*, *K*, *m*, and *l* represent the radial shifting parameter, petal frequency, exponent, and topological charge, respectively. In a spiral PZP with polygon-like focused intensity distributions, the side number of the polygon is equal to the product of *K* and *m*, which is an integral number. To obtain the binary PZPs, the transmittance function is binarized as

(3) $$\text{BT}\left(r,\theta \right)=\left\{\begin{array}{l} 1\text{ imag}\left[T\left(r,\theta \right)\right]\text{ > 0 }\\ 0\text{ imag}\left[T\left(r,\theta \right)\right]\text{ < 0} \end{array}\right.$$

where imag[*T*(*r*,*θ*)] is the imaginary part of *T*(*r*,*θ*).

**Figure 4:**
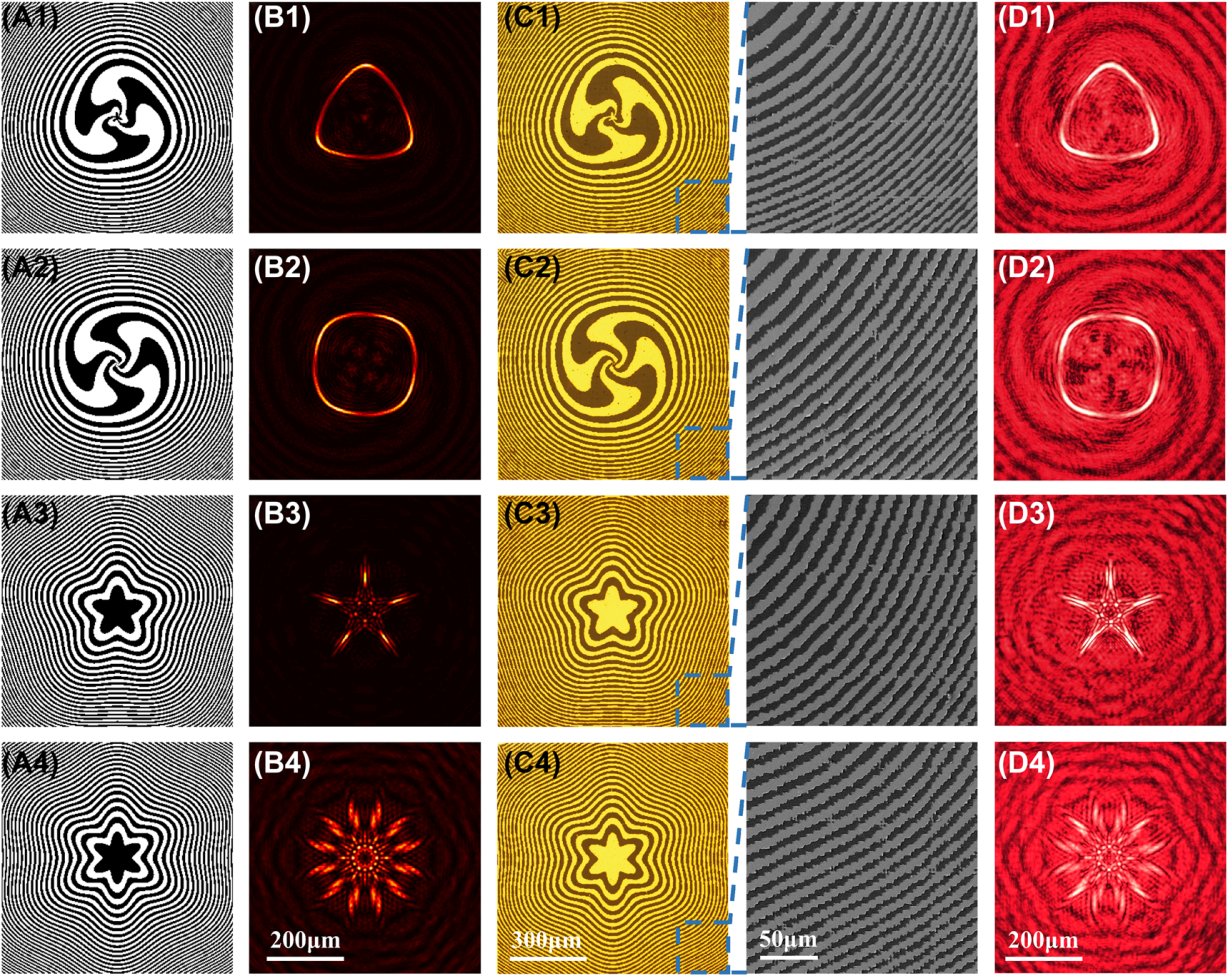
Demonstration of the PZPs. (A1–4) Examples of PZPs (600 × 600 px) with focused patterns: Triangle-like, square-like, and star-like with five arms and twelve arms, respectively. (B1–4) simulated intensity distributions at the focal planes of the calculated PZPs. (C1–4) microscopy images and local enlarged SEM images of the fabricated PZPs. (D1–4) corresponding measured intensity distributions at the focal planes.

Figure 4A1–2 illustrate calculated binary amplitude–type spiral PZPs with *K* = 1.5, *m* = 2, *α* = 0.07, *f* = 7 mm, *r*_0_ = 0.3 *R*, and *l* = 3 and *K* = 4, *m* = 1, *α* = 0.01, *f* = 7 mm, *r*_0_ = 0.3*R*, and *l* = 4, respectively. Figure 4A3–4 depict PZPs with annular petal-like zones with *K* = 5, *m* = 1, *α* = 0.05, *f* = 10 mm, *r*_0_ = 0, and *l* = 0 and *K* = 6, *m* = 1, *α* = 0.05, *f* = 10 mm, *r*_0_ = 0, and *l* = 0, respectively. The other parameters of the calculated PZPs are *λ* = 633 nm and *R* = 900 μm, and each PZP contains 600 × 600 px. Figure 4B1–4 present the simulated intensity distributions at the focal planes of the four PZPs, with focused patterns that are triangle-like, square-like, and star-like with five and twelve arms, respectively. The fabricated PZPs stitched with 400 element structures on Au film are illustrated in Figure 4C1–4, and their corresponding measured intensity distributions at the focal planes are presented in Figure 4D1–4. The experimental results are consistent with the simulated results, indicating that the proposed splitting–stitching method can be extended to fabricate various types of high-quality zone plates.

### 3.4 Fabrication of FZP arrays

**Figure 5:**
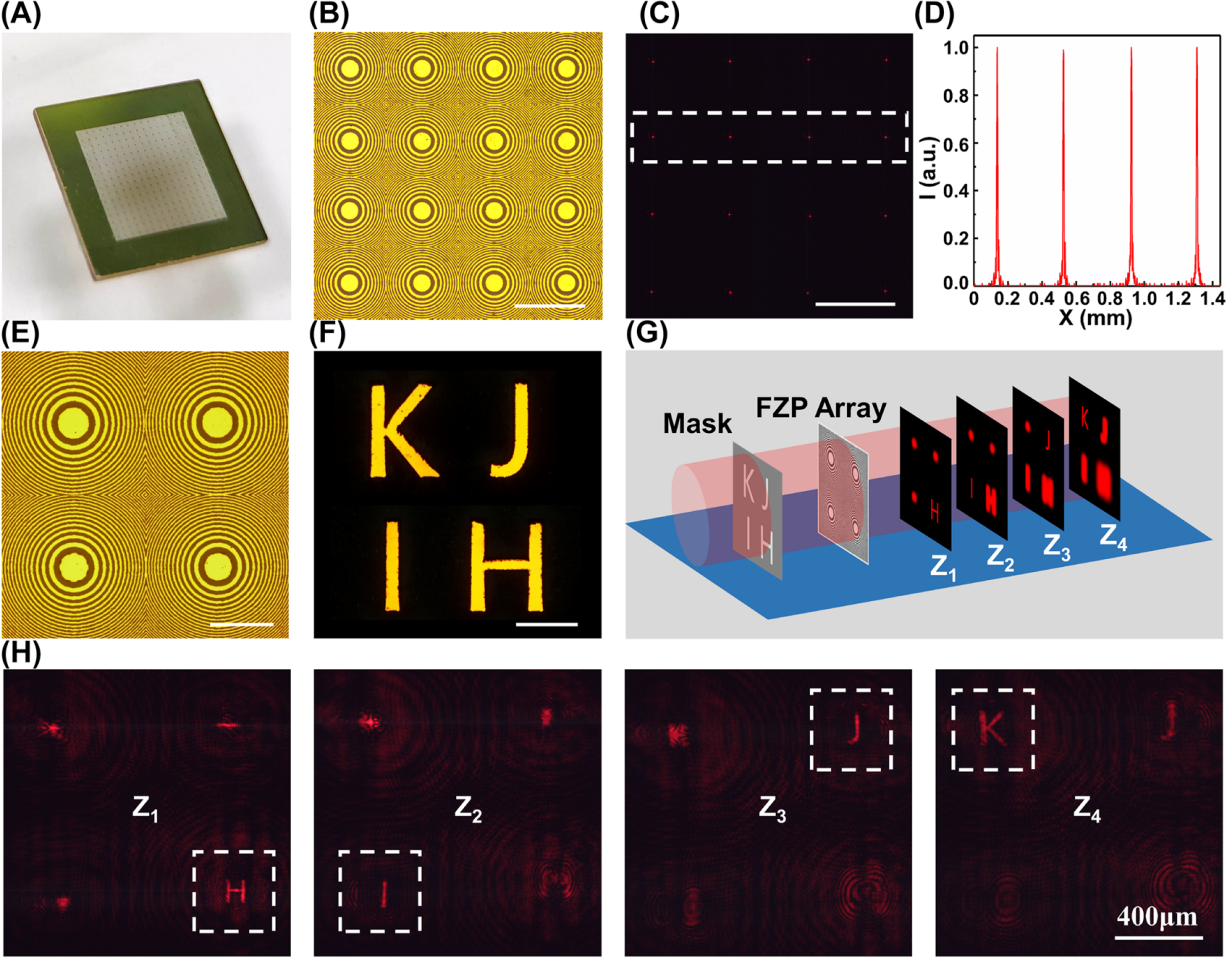
Fabrication of large-scale FZP arrays. (A) Photograph of a 16 × 16 square-arranged FZP array (*f* = 4 mm) with an overall size of 6.48 × 6.48 mm. (B) Magnification microscopy image of a 4 × 4 array. (C) Corresponding measured intensity distribution of a 4 × 4 multifocal array. (D) normalized intensity profile plotted along the central cross section of the focal spots in the white dashed box. (E) microscopy image of a 2 × 2 FZP array consisting of four FZPs with a focal length of 12, 13, 14, and 15 mm, respectively. (F) Microscopy image of the laser-fabricated mask captured using transmittance mode. (G) imaging separation of the microletter hollow patterns on a mask using the 2 × 2 FZP array. (H) Captured images at different focal planes of the FZP array. The white dashed boxes indicate the corresponding imaged microletters.

Apart from fabricating single zone plates, the proposed method was also employed to rapidly fabricate large-scale FZP arrays. Figure 5A presents a square-arranged FZP array with an overall size of 6.48 × 6.48 mm and consisting of 16 × 16 FZPs (*f* = 4 mm), with each FZP containing 270 × 270 px. The magnification microscopy image of a 4 × 4 array and the corresponding measured multifocal arrays are presented in Figure 5B and C, respectively. Figure 5D depicts the normalized intensity profile plotted along the central cross section of the focal spots in the white dashed box in Figure 5C. The uniform morphological and optical characterization results indicate that the splitting–stitching method is an effective technique for fabricating large-scale FZP arrays. Furthermore, because of the flexibility of the optical modulation based on the SLM, the optical properties of a single FZP unit in an array can be conveniently adjusted when processing. Figure 5E presents a 2 × 2 FZP array consisting of four FZPs with a focal length of 12, 13, 14, and 15 mm. Each FZP contains 600 × 600 px. Such a FZP array with different focal lengths can be used to realize the imaging separation of objects, which can be applied to the dividing disposal of optical signals. Figure 5F presents a foil mask containing four hollow patterns forming the microletters “H,” “I,” “J,” and “K”. As illustrated in Figure 5G, different microletters on the mask can be imaged separately at different focal planes using the 2 × 2 FZP array. In Figure 5H, the “H,” “I,” “J,” and “K” patterns marked by the white dashed boxes are imaged clearly in sequence along the optical axis.

## 4 Conclusions

In conclusion, a splitting–stitching method based on holographic femtosecond laser pulses was used to fabricate cross-scale ultrathin FZPs on Au film with high processing efficiency. Different sizes of FZP were split into a series of element patterns, which were printed on the metal film in sequence by dynamically shaping the femtosecond pulses. The stitched element structures constituted the complete FZPs, which possessed a broadband focusing and imaging capability in the visible spectrum. Benefiting from the flexibility of optical modulation, other forms of zone plates (axial multifocal zone plates, different types of PZPs, and FZP arrays with uniform or different focal lengths) were designed and successfully fabricated using the proposed method. The high agreement between the focusing simulations and experimentally measured results revealed the high processing quality of the zone plates, demonstrating the great potential of the proposed method for efficiently fabricating functional planar lenses of high quality.

## Supplementary Material

Supplementary Material Details
