# Supplementary material for: High efficiency and scalable fabrication of fresnel zone plates using holographic femtosecond pulses
Source: Nanophotonics. 2022 May 24;11(13):3081–91. doi: 10.1515/nanoph-2022-0112 (PMC11501355; doi:10.1515/nanoph-2022-0112)
Supplement: Supplementary file 1 — Supplementary Material Details [file j_nanoph-2022-0112_suppl.docx]

**Supplementary Material**

**High Efficiency and Scalable Fabrication of Fresnel Zone Plates Using Holographic Femtosecond Pulses**

Zhipeng Wang^1^, Lan Jiang^1,2^, Xiaowei Li*^1^, Shuai Gao^1^, Shipeng Zhou^1^, Yang Liu^1^, Lingling Huang^3^, Jiangang Lu^4^ and Jiangang Yin^4^

*^1^ Laser Micro/Nano Fabrication Laboratory,* *School of Mechanical Engineering, Beijing Institute of Technology, Beijing 100081, China*

*^2^ Beijing Institute of Technology Chongqing Innovation Center, Chongqing, 401120, China*

*^3^ School of Optics and Photonics, Beijing Institute of Technology, Beijing 100081, China*

*^4^ Han’s Laser Technology Centre, Shennan Ave No. 9988, Nanshan District, Shenzhen, Guangdong, 518057, China*

*E-mail: lixiaowei@bit.edu.cn

**1.** **Caulated focusing efficiency of the FZP with *f* = 12 mm**


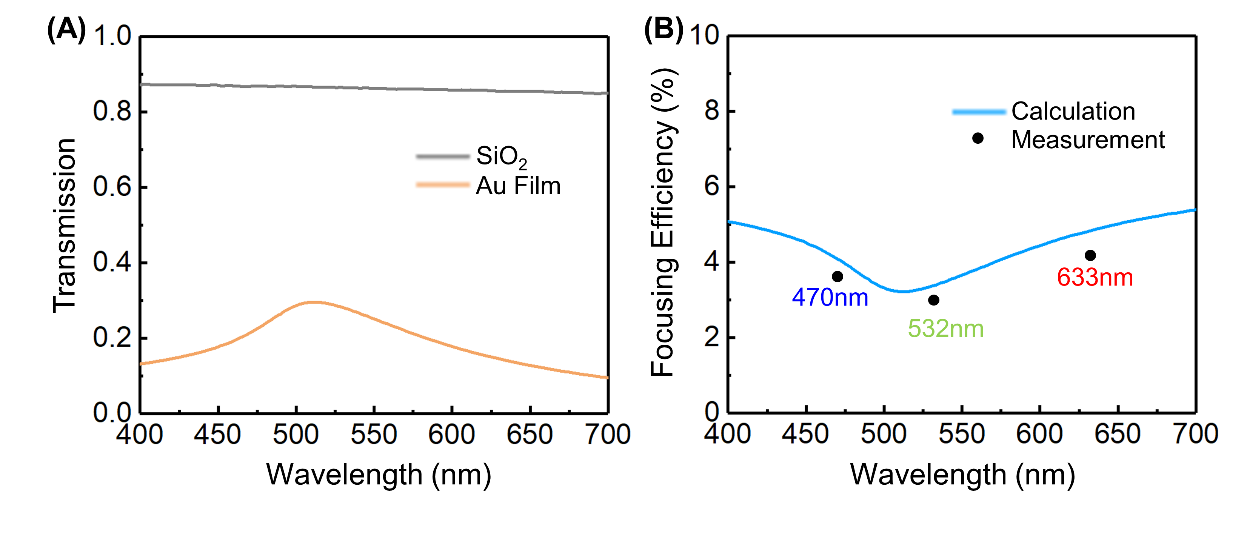


Figure S1. Calculation of focusing efficiency of the FZP with *f* = 12 mm based on the transmittance of materials. (A) Measured optical transmittances of SiO_2_ substrate and Au film in the visible spectrum (400−700 nm). (B) Calculated focusing efficiencies of the FZP at different wavelengths based on the measured results of transmittance. The measured results at the wavelengths of 470, 532, and 633 nm are also provided for comparison.

**2. Measured intensity distributions of the main focus and subsidiary foci**


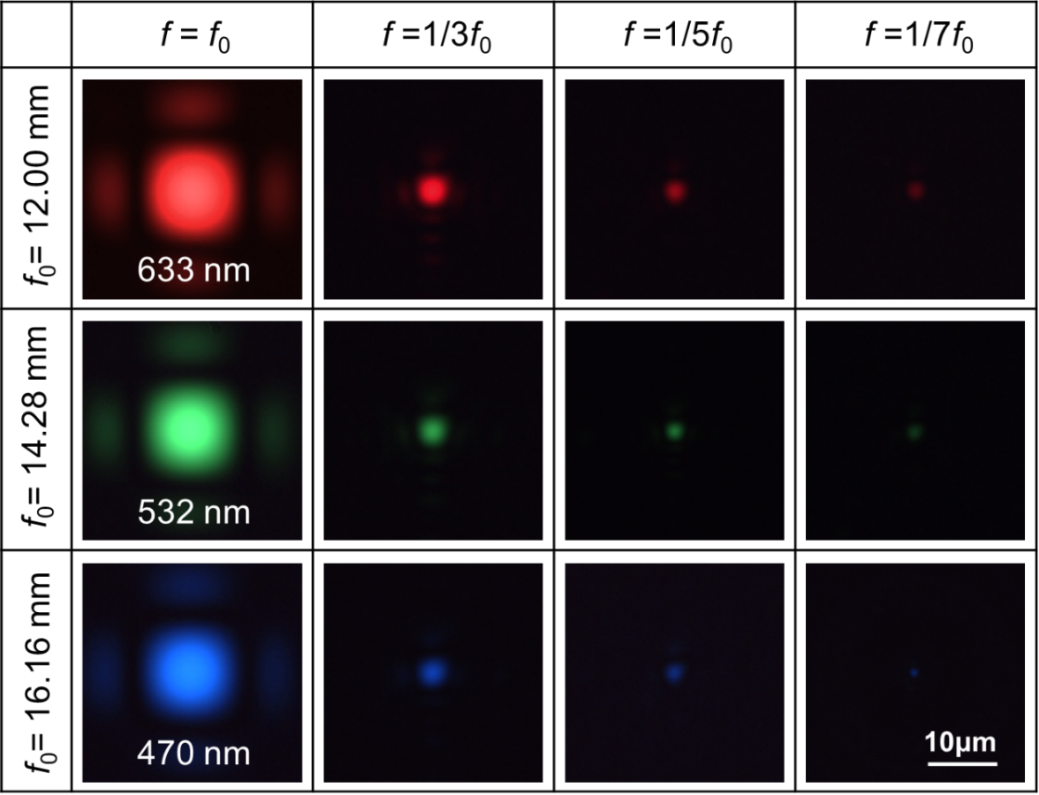


Figure S2. Intensity distributions of the main focus and subsidiary foci along the optical axis of the optical field diffracted by the fabricated FZP with *f* = 12 mm under laser beam irradiation of 470, 532, and 633 nm.

**3. The design process of** **the axial multifocal zone plate**

A 600 × 600-px square zone was divided into 48 sectors with the same center angle. The 48 sectors were then divided into three parts, forming three masks (Figure S3A1–3). Each mask contained 16 sectors with an interval of 15°. The sectors in the three masks were then filled with FZP patterns with focal lengths of 15, 17.5, and 20 mm at a laser wavelength of 633 nm (Figure S3B1–3), and the obtained sectored zone plates are illustrated in Figure S3C1–3. Figure S3D1–3 depicts the intensity distributions at the focal planes, longitudinal intensity distributions in the XZ planes, and normalized profiles along the Z-axis of the diffracted optical fields through the three sectored zone plates. Although the sectored zone plates only constitute a third of the corresponding FZPs, they retain the focusing capability. Finally, the multifocal zone plate is obtained by superposing the three sectored zone plates.


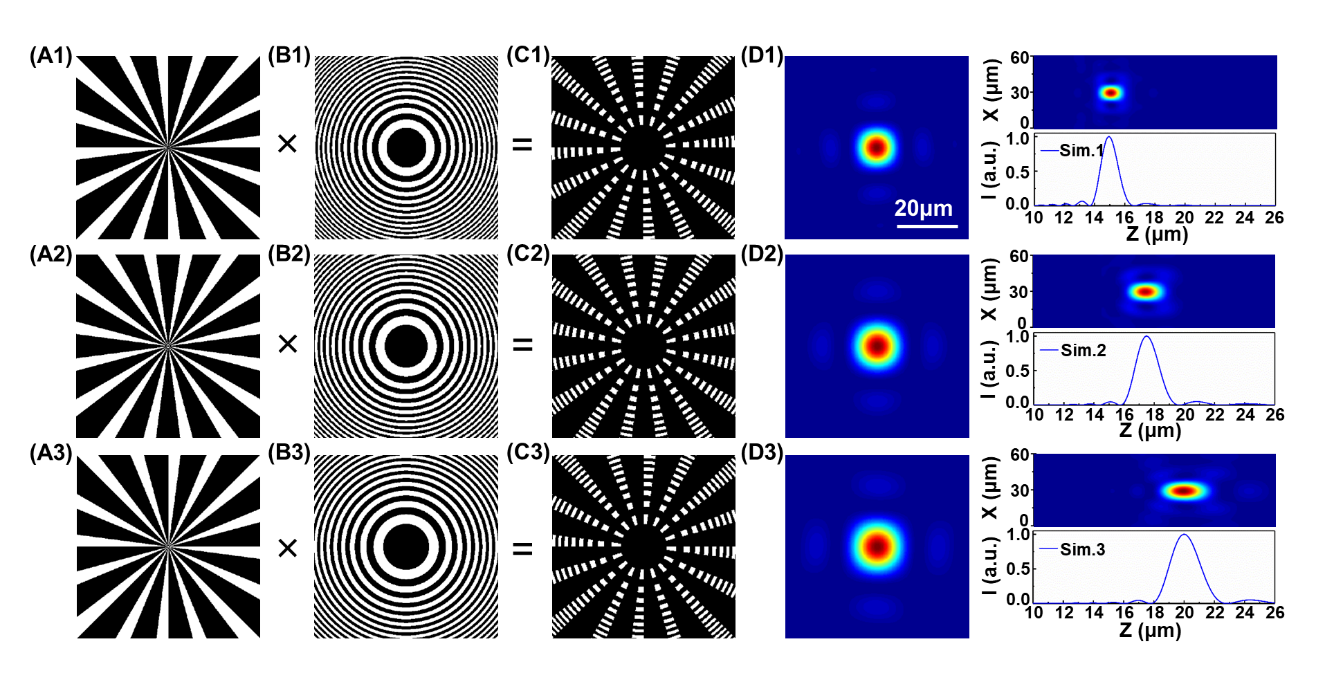


Figure S3. Design process of the axial multifocal zone plate. (A1–3) Masks consisting of 16 sectors with an interval of 15°. (B1–3) Calculated FZP patterns with focal lengths of 15, 17.5, and 20 mm, respectively. (C1–3) Sectored zone plates formed by filling the corresponding masks with FZPs. (D1–3) Simulated intensity distributions at the focal planes, longitudinal intensity distributions in the XZ plane, and normalized intensity profiles along the Z-axis of the diffracted optical fields through the sectored zone plates.

**4. Measured imaging results of the axial multifocal zone plate**


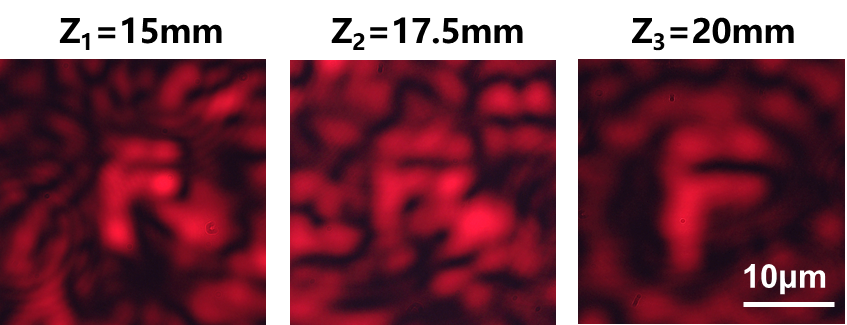


Figure S4. Captured images of the letter “F” at the three focal planes (Z_1_ = 15 mm, Z_2_ = 17.5 mm, and Z_3_ = 20 mm) of the fabricated multifocal zone plate.
